# The Social Validity of Telepractice among Spanish-Speaking Caregivers of English Learners: An Examination of Moderators

**DOI:** 10.5195/ijt.2017.6227

**Published:** 2017-11-20

**Authors:** LISA FITTON, KRISTINA N. BUSTAMANTE, CARLA WOOD

**Affiliations:** 1 SCHOOL OF COMMUNICATION SCIENCE & DISORDERS, FLORIDA STATE UNIVERSITY, TALLAHASSEE, FL, USA

**Keywords:** Bilingualism, Minority language, Telehealth, Telepractice

## Abstract

The purpose of the present paper was to examine the social validity of telepractice as a service delivery model for Spanish-speaking families of English learners. Quantitative survey methodology was employed to examine 79 caregivers’ opinions regarding telepractice and to obtain background information about participants’ home environments. Findings revealed that approximately 46% of the participant sample reported being interested in their children receiving services via telepractice. Caregivers reported limited familiarity with telepractice as an option, but were likely to express interest if their child had an identified speech or language disorder or if they were interested in increased access to Spanish language support for their children. In conclusion, although telepractice is not universally accepted among Spanish-speaking families, it appears to be a promising service delivery model. It is recommended that service providers offer thorough information and address common myths when considering telepractice as a service delivery model for families.

In the United States, 21% of school-age children speak a language other than English at home (U.S. Census Bureau, 2015). These children learning English in school are referred to as English language learners or English learners (ELs) and are part of the substantial minority language speaker population observed worldwide (Wiley, Garcia, Danzig, & Stigler, 2014). In 2012, over 60% of speech-language pathologists in the United States reported having at least one EL on their caseloads (ASHA). As attention to this linguistic minority grows, an increasing number of educators and service providers are encountering barriers that obstruct effective service delivery to ELs. The purpose of the present study was to evaluate the social validity of telepractice, a promising approach proposed to address some of these challenges.

## BARRIERS TO HIGH QUALITY SERVICE DELIVERY FOR ELS

In the United States, there are policies in place designed to mandate culturally and linguistically appropriate services for all children (ASHA, 2016b, IDEA, 2004). However, barriers create challenges to universal implementation of this directive, particularly among minority language speakers. We will review common barriers including gaps in knowledge, linguistic mismatch, distance to bilingual services, and the shortage of bilingual service providers. Many educators and service providers have reported they do not feel they have adequate preparation and training to distinguish the developmental differences between ELs and monolingual language learners, and are less comfortable working with linguistic minorities compared to working with culturally and racially diverse populations (e.g., Bedore & Peña, 2008; Guiberson & Atkins, 2012; Jackson, Leacox, & Callender, 2010).

According to the ASHA Code of Ethics, service providers who feel unqualified to support individuals from different linguistic backgrounds should refer these individuals to other professionals (ASHA, 2016). The rationale for this referral requirement can be found by considering the compounding problems of a linguistic mismatch between professionals and ELs’ caregivers, particularly when caregivers have limited English proficiency. Caregiver-professional linguistic differences can lead to decreased caregiver input (Arias & Morillo-Campbell, 2008) and reduced caregiver knowledge regarding their child’s status (Buysse, Castro, West, & Skinner, 2004). Scheduling appointments, expressing concerns, and obtaining information about their children’s therapy are more difficult with linguistic barriers present, and consequently caregivers can become less involved in their children’s education (Arias & Morillo-Campbell, 2008). Given that caregiver involvement in children’s academics is critical to accurate identification of language impairment and progress in therapy (Justice & Ezell, 2000; Roberts, Jurgens, & Burchinal, 2005), poor access to bilingual services can negatively affect child outcomes.

Employing an interpreter is recommended to facilitate communication when a linguistic mismatch occurs and a qualified service provider is not accessible, but this practice does not resolve all the barriers to effective service delivery (ASHA, 2016). Even when interpreters provide open communication between families and providers, knowledge of multilingual language development is necessary to provide appropriate services to ELs. Accurate assessment and effective intervention require familiarity with the impact of (1) low English proficiency, (2) knowledge of another language, and (3) different sociocultural backgrounds on language and literacy acquisition in ELs (Paradis, Genesee, & Crago, 2011). Limited service provider knowledge can lead to over- or under-identification of ELs as having speech or language impairment (Bedore & Peña, 2008) and to delayed progress (Kohnert, 2010).

### SHORTAGE OF BILINGUAL SERVICE PROVIDERS

Ideally, clients would be matched with skilled bilingual service providers for efficiency and quality (ASHA, 2016; Kohnert, 2010); however, relative to the number of ELs in the United States, there is a shortage of bilingual speech-language pathologists (ASHA, 2012). With limited options for caregivers of ELs who do not have access to bilingual practitioners, families may be asked to drive substantial distances to see bilingual providers or choose to see English-only providers. Because of the high prevalence of poverty among families with low English proficiency (Cosentino de Cohen, Deterding, & Chu Clewell, 2005), costs related to travel to access services can further inhibit families’ participation and access to high quality services (Hernandez, 2004).

### ADDRESSING BARRIERS THROUGH TELEPRACTICE

In response to the limited service delivery options available to linguistically-diverse populations, telepractice has emerged as a promising strategy for increasing access to preferred services (Pham, 2012; Theodoros, 2012; Tucker, 2012). Endorsed by ASHA as an ethical option, telepractice is “the application of telecommunication technology to deliver professional service at a distance by linking clinician to client, or clinician to clinician for assessment, intervention, and/or consultation” (ASHA, n.d.). Through videoconferencing and other continually-evolving technologies, telepractice can offer synchronous interaction when service providers and children are in separate locations.

### EVIDENCE-BASED PRACTICE: TELEPRACTICE

Before adopting telepractice for young ELs, it is necessary to examine external empirical evidence, clinical expertise and expert opinion, and caregiver perspectives as they relate to telepractice (ASHA, n.d.). Telepractice directly addresses accessibility barriers and as a result may be associated with fewer client absences and greater intervention frequency than when services are compared to in-person sessions (Baharav & Reiser, 2010; Forducey, 2006; Kobak et al., 2011; Vismara, Young, & Rogers, 2012). However, for telepractice to be considered best practice it must produce satisfactory outcomes with evidence from empirical research, approval of clinical experts, and social validity among caregivers.

Empirically, a growing body of evidence supports the efficacy of telepractice. Emerging research suggests that telepractice and in-person service delivery produce comparable outcomes (e.g., McCullough, 2001; Pham, 2012). In a study designed to compare language assessment conducted in-person to that conducted via telepractice, no significant differences were noted between test scores (Waite, Theodoros, Russell, & Cahill, 2010). In addition, telepractice has been shown to benefit intervention practice through reduced costs and increased access to services, yielding comparable outcomes to in-person practice (e.g., Grogan-Johnson et al., 2011).

Service providers have generally responded positively to telepractice use (e.g., Tucker, 2012). Reduced absence, continued gains in targeted skills, and increased access to services have been cited as contributors to clinician’s acceptance of telepractice (Forducey, 2006; McConnochie et al., 2005; Theodoros, 2012). However, service providers have also identified limitations to widespread telepractice implementation, including concerns regarding technology cost and reliability, lack of physical contact, and reimbursement barriers (Tucker, 2012), suggesting feasibility and acceptance of telepractice is disputable in some areas.

### GAPS IN THE LITERATURE

Information about caregiver perspectives is less widely-documented than the first two components of evidencebased practice. Several pilot studies suggest that caregivers are satisfied with telepractice after receiving telepractice intervention (Baharav & Reiser, 2010; Kobak et al., 2011; Pham, 2012), but these studies provide only retrospective evidence of family opinions. For service providers who are considering recommending telepractice to caregivers of ELs, the opinions of families with no prior experience with the service delivery model are more beneficial. Critically, families with no prior experience with telepractice may be more likely to be misinformed regarding how this service delivery model is conducted. Lack of information or acceptance of common myths surrounding telepractice (e.g., telepractice is illegal; Geurin, 2009) may influence families’ service delivery preferences, making them less likely to want their children to participate in telepractice.

Families’ opinions of the service delivery model can directly inform future developments in telepractice-delivered therapy (see Wolf, 1978). Additional examination of both general opinions and potential moderating factors of these opinions is needed to evaluate the social validity of telepractice for Spanish-English speaking children. The motivation for the present study is to help practitioners better understanding the beliefs of ELs’ families in order to apply evidence-based practice more comprehensively. The research aims to examine the social validity of telepractice among caregivers of Spanish-speaking ELs in the United States. Spanish-speaking caregivers were focused on because the United States’ most populous linguistic minority is Hispanic (U.S. Census, 2014). Furthermore, only 68.4% of Hispanic individuals ages five and older speak English ‘very well’ (U.S. Census, 2014). The study was designed to address the following:

1. Is telepractice a socially-valid service delivery model for families of Spanish-English speaking ELs?
2. What factors moderate interest in telepractice for families of Spanish-English speaking ELs?

## METHOD

A survey was constructed to obtain information about telepractice as a service delivery model for caregivers’ children. Upon receipt of informed consent, participants completed the survey in their preferred language. To maximize construct validity, the instrument underwent pilot sampling and was then refined based on participant feedback and item analysis. Participants were caregivers of Spanish-speaking children recruited from schools and migrant education programs in northern Florida, Michigan, and Illinois. All procedures were approved by the Human Subjects Committee at Florida State University.

### SURVEY PILOT AND REFINEMENT

The pilot version of the instrument was 17 pages and consisted of 56 items written in both Spanish and English. Question format included rating scales, yes-no, multiple choice, and open-ended questions to examine the completeness and precision of response by different item types. Questions pertaining to demographics, family language use, and caregiver/child fluency in English and Spanish were included in accordance with best practice for researchers to specify the language dominance of bilingual individuals included in their samples (U.S. Dept. of Education IES WWC, 2013). Caregiver opinions regarding bilingualism, Spanish, and English use were targeted to examine possible relations between telepractice and access to bilingual services (e.g., Pham, 2012). Items focusing on child educational experiences and accessibility to educational services were included to assess general accessibility as a moderator of family interest in telepractice (Forducey, 2006; Vismara et al., 2012). Finally, the survey included questions related to caregiver knowledge and interest in telepractice, and to family’s access and competence with technology, which have been cited as common barriers to telepractice implementation (ASHA, n.d.; Geurin, 2009).

Piloting occurred with 34 caregivers who reported speaking primarily Spanish to their children. The participants were recruited using the following eligibility criteria: (a) the participant was the caregiver of at least one child who was between the ages of 0 and 8 years, (b) the participant’s child was consistently exposed to some Spanish at home, and (c) the family lived in the United States. Children were not required to have any exposure to English.

The investigators revised the survey in response to pilot participants’ responses and individual feedback. Based on item-analysis, investigators retained reliable questions and edited or eliminated questions that may have been confusing or misworded. Refinement from pilot participant feedback focused on: (a) clarity of the wording of the questions; (b) brevity, shortening the questionnaire; and (c) parallelism of questions. Investigators removed unreliable open-ended questions and consolidated parallel questions. Items that were skipped consistently in the pilot were removed (e.g. “What is your child’s fluency level for reading in Spanish?”).

The final instrument included 37 items and was 10 pages long. The structure of the instrument was similar to that of the pilot instrument, including rating scales, yes-no, multiple choice, and open-ended questions. Content areas were also the same, focusing on demographic information, family language use, fluency in English and Spanish, opinions regarding Spanish and English use, child educational experiences, obstacles encountered in the child’s education, knowledge of and interest in telepractice, and access to and competence with technology. There were two versions of the survey, one in Spanish and one in English, to allow participants to respond in their preferred language. Surveys were hand-delivered or mailed to potential participants and included a stamped return envelope.

### ANALYSES

Descriptive statistics regarding questionnaire response and completion rates were first examined. To reduce the risk of measurement bias related to the construction of the questionnaire, any items that had a missing data rate of 25% or greater were excluded from subsequent analysis. Descriptives appropriate to the data type, including frequencies, means, and standard deviations, were then obtained for respondent, child, and family characteristics to attain information about the participant sample.

To ascertain the overall social validity of telepractice, frequencies and modes for items targeting caregiver interest in and knowledge of telepractice were examined. Next, to prepare for moderator analyses, composite indices were computed from multiple survey items designed to target the same underlying construct. The construction of these indices was based on prior example of aggregating similar items to represent a single construct (e.g., Montrul, 2012).

To create each composite, all items to be included in the composite were first z-scored to create comparable scaling for the composite (Cohen, Cohen, West, & Aiken, 2003). The z-scored items were then aggregated through averaging. This approach was selected to reduce the impact of missing data, so that missing responses did not result in skewing of the composite score, and to weight each survey item equally within its composite. The composite indices included: caregiver Spanish fluency, caregiver English fluency, child Spanish fluency, child English fluency, caregiver’s value of culture, family access to technology needed to receive telepractice services, caregiver competence with technology needed to receive telepractice services, and parent belief in telepractice myths. The items included in each composite are listed in Table 1.

To identify potential moderators of caregivers’ interest in telepractice, bivariate relations between background factors and participants’ reported interest in telepractice were examined by obtaining non-parametric correlation coefficients. Although both Kendall’s tau and Spearman’s rho are both considered acceptable for obtaining non-parametric correlation estimates, Kendall’s tau was selected because it generally yields more conservative estimates and is considered more robust to nonnormality (Croux & Dehon, 2010). To examine relations between dichotomous background variables and reported interest in telepractice more closely, cross tabulation with chi-square testing was also completed. Background factors of interest as potential moderators were: (a) caregiver/child language fluency in English and Spanish (U.S. Dept. of Education IES WWC, 2013); (b) caregiver value of culture (e.g., Pham, 2012); (c) access to and competence with technology (ASHA, n.d.); (d) belief in telepractice myths (Geurin, 2009); and (e) need for telepractice-delivered services, as measured by whether or not the child was diagnosed with a speech or language disorder and caregiver interest in the child receiving Spanish language support (Forducey, 2006; Vismara et al., 2012).

Finally, to determine how much of the variability in caregivers’ interest in telepractice could be predicted by their other questionnaire responses, multiple logistic regression was conducted. Caregiver report of interest in telepractice was included as the outcome. Background factors were included as predictors in the model only if they were revealed to relate significantly to interest in telepractice during bivariate testing.

## RESULTS

### DESCRIPTIVE RESULTS

Responses from the final instrument were obtained from 79 Spanish-speaking caregivers. Of the 100 surveys hand-delivered by service providers and educators in Florida, 41 were completed. Approximately 125 surveys were delivered by mail to interested individuals in Illinois and Michigan and 20 were mailed back to the investigators. An additional 18 surveys from respondents who declined to report their current state of residence were delivered to the investigators. The overall response rate for the invited individuals was 35.1%.

Of the returned questionnaires, 44.3% were fully completed. Most participants (98.7%, *n* = 78) responded to all demographic questions, and no patterns were observed between demographics and missing data. Four total items had response rates below 75% and were consequently excluded from subsequent analyses. All four of these items were follow-up questions (e.g., “please explain” following the primary question of “ideally, who would deliver services to your child?”) and were not considered central to the content of the questionnaire. Outside of these four items, the most frequently skipped items were the child’s date of birth (missing 21.5%, *n* = 17) and items designed to examine caregivers’ belief in telepractice myths (missing 22.8%, *n* = 19). In place of the child’s date of birth, most of the caregivers wrote in the child’s age. Most participants (87.3%, *n* = 69) completed at least 75% of the questionnaire.

**Figure 1:**
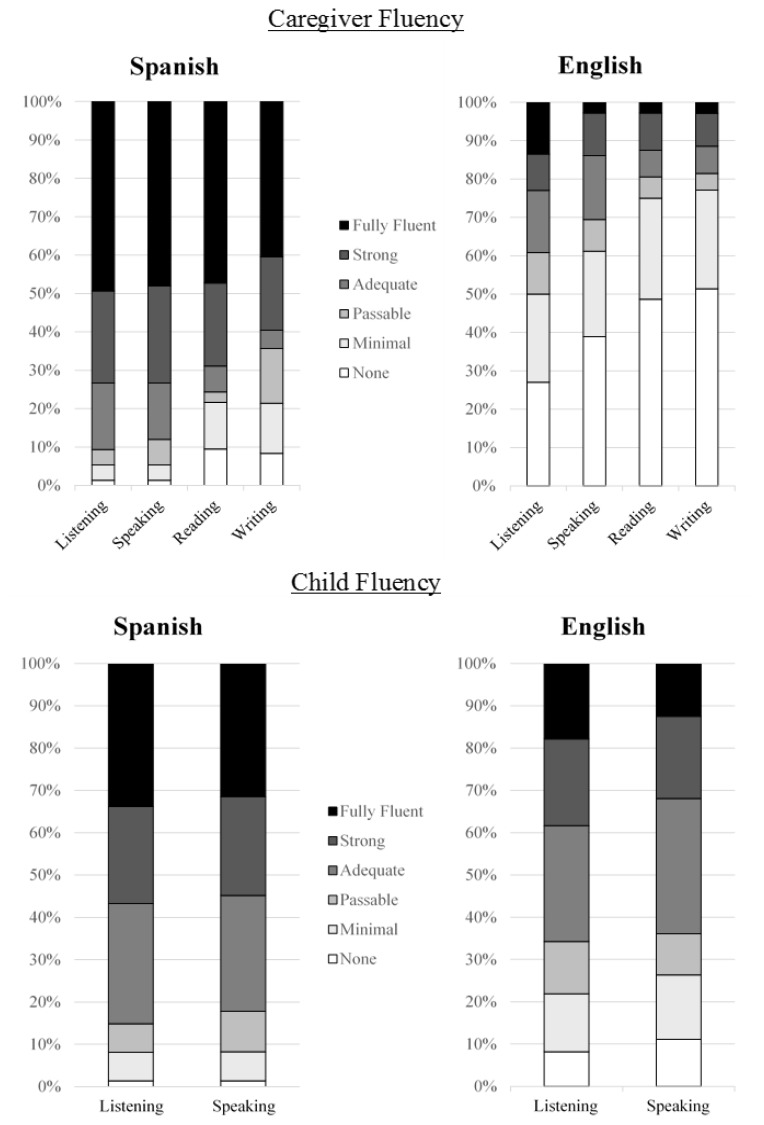
Caregiver and child fluency in English and Spanish.

Respondents identified themselves as the parent of an EL in 98.7% (*n* = 77) of cases. The remaining respondent identified herself as the grandparent of an EL. All respondents reported speaking at least some Spanish at home (*n* = 76) and 32.9% (*n* = 25) reported also using some English. Over half of families (67.1%, *n* = 51) reported Spanish-only home environments. Figure 1 provides the self-reported fluency levels of caregivers and of their children. Of the participants who reported their educational backgrounds, more than half indicated that they did not attend high school. An additional 24% of caregivers reported starting high school without graduating.

The children identified as ELs were between the ages of 1 year, 9 months to 18 years, with an average age of 7 years, 8 months (*n* = 74). When asked about their children’s speech and language development, 27.8% (*n* = 22) of caregivers indicated that their child had been diagnosed with a speech or language disorder. Of the remaining caregivers, 62.0% (*n* = 49) reported that their child had no speech or language diagnosis, 5.1% (*n* = 4) were unsure, and 5.1% did not respond (*n* = 4). Additional demographic information is reported in Table 2.

### SOCIAL VALIDITY OF TELEPRACTICE

**Figure 2:**
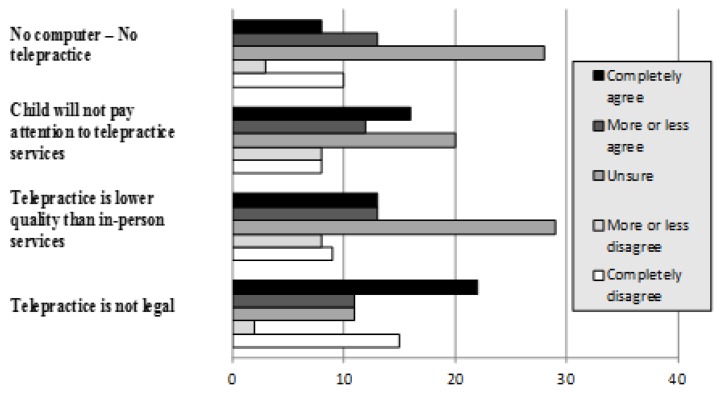
Caregiver opinions regarding telepractice myths.

Few participants indicated they had any knowledge of telepractice prior to participation in the study (3.8%, *n* = 3). However, given the brief definition of telepractice, 45.6% (*n* = 31) of respondents stated they were interested and 54.4% (*n* = 37) stated they were not interested. Participants expressed mixed agreement with each of the telepractice myths (see Figure 2).

### MODERATORS OF TELEPRACTICE INTEREST

Correlational findings revealed that caregiver fluency in English, child fluency in both English and Spanish, caregiver value of culture, family access to and competence with technology, and belief in telepractice myths were not significantly related to reported interest in telepractice. However, caregiver fluency in Spanish, child diagnosis of a speech or language disorder, and caregiver interest in their child receiving Spanish language support were significantly associated with interest in telepractice service delivery. See Table 3 for Kendall’s tau correlation coefficients.

Caregivers who reported stronger Spanish fluency levels more commonly reported being interested in telepractice (τ = .31, *p* < .001). Those who had children with a diagnosed disorder also expressed interest in telepractice significantly more often than caregivers of children without a speech or language disorder, evidenced by correlational findings (τ = .47, *p* < .001) and cross-tabulation chi-square testing: χ^2^(2, *n* = 65) = 19.01, *p* < .001. Of the 18 caregivers who reported that their child had a speech or language disorder, 16 indicated interest in telepractice. Of the remaining 43 caregivers, 31 indicated they were not interested in telepractice and 12 indicated they would be interested in telepractice service delivery.

Caregivers who were interested in their child receiving Spanish language support were also significantly more interested in telepractice as a service delivery model than those who did not express interest in Spanish support, evidenced by both correlational findings (τ = .62 *p* < .01) and cross-tabulation: χ^2^(1, *n* = 61) = 23.55, *p* < .001. Of the 32 families who wanted Spanish language support for their children, 23 reported they would be interested in receiving services via telepractice. Nearly all (*n =* 26, 89.66%) of the 29 caregivers who were not interested in Spanish language support were similarly uninterested in telepractice.

One post-hoc exploratory test was conducted to examine caregiver report of difficulty accessing services for their child. A cross-tabulation chi-square test revealed a significant difference, χ^2^ (1, *n* = 62) = 25.04, *p* < .001. Caregivers who reported difficulty obtaining access to services (*n* = 14) unanimously indicated that they would be interested in their child receiving telepractice services.

Multiple logistic regression was conducted to examine how family interest in telepractice was predicted by caregiver fluency in Spanish, child diagnosis of a speech or language disorder, and caregiver interest in their child receiving Spanish language support. A significant overall result was found for the initial three-predictor model χ^2^ (3) = 32.78, *p* < .001. A pseudo *R*^2^ value of .604 was obtained, suggesting a moderately strong relation between the predictors and reported interest in telepractice. The Hosmer and Lemeshow Test was not significant χ^2^ (6) = 6.00, *p* = .423, indicating an acceptable model fit. Two of the predictors, however, exhibited evidence of multicollinearity; caregiver fluency in Spanish and caregiver interest in their child receiving Spanish support were significant correlated (τ = .31, *p* = .007) and did not both uniquely contribute to predicting family interest in telepractice services (see Table 4). Because interest in receiving Spanish support was more strongly related to family interest in telepractice than caregiver fluency in Spanish, caregiver Spanish fluency was excluded from the model.

The final model predicting family interest in telepractice included two predictors: child diagnosis of a speech or language disorder and caregiver interest in receiving Spanish language support. The omnibus test of the model was significant χ^2^ (2) = 34.40, *p* < .001, and yielded a pseudo *R*^2^ of .585. The Hosmer and Lemeshow Test for the model was not significant χ^2^ (2) = 1.51, *p* = .471. Both predictors significantly contributed to the likelihood that caregivers would express interest in telepractice. Families of children who had been diagnosed with a speech or language disorder were more likely to express interest in telepractice services, and those who expressed interest in receiving Spanish language support were also more likely to be interested in telepractice. Both of these predictors uniquely contributed to family interest in telepractice.

## DISCUSSION

The purpose of the present study was to evaluate the social validity of telepractice as a potential service delivery model for Spanish-speaking families of English learners, and to identify moderators of families’ interest in telepractice. Quantitative survey methodology was employed to obtain feedback from a diverse sample of Spanish-speaking caregivers of English learners. The survey was designed to elicit caregivers’ opinions regarding telepractice as a service delivery model, and to obtain information regarding potential factors relating to families’ interest in telepractice as a desired option for their children.

### THE SOCIAL VALIDITY OF TELEPRACTICE

Our findings indicate that Spanish-speaking caregivers’ interest in telepractice for their children is currently limited to specific sub-groups of caregivers, which were represented by 46% of our sample. Over half of the caregivers surveyed indicated that they would not be interested in their child receiving any educational support via telepractice. Although this finding is surprising when considered next to prior work that has suggested telepractice is a positive experience for many Hispanic caregivers (e.g., Vismara et al., 2012), the background characteristics of the present sample offer reasonable explanation. Nearly all caregivers of children with a diagnosed speech or language disorder indicated that they would be interested in telepractice services. Caregivers of children without a diagnosis were divided, with less than a third of these caregivers expressing interest in telepractice. These findings suggest that caregivers are more likely to be interested in unfamiliar service delivery options when their children had a confirmed diagnosis and perhaps motivation based on an immediate need for services, as is the case with ELs with a speech or language disorder. This conclusion was bolstered by evidence that caregivers who had experienced challenges in obtaining appropriate services for their children were more likely to express interest in telepractice than those who did not report difficulty accessing services.

Caregivers who were interested in Spanish language support for their children more frequently reported being interested in telepractice, and reported higher levels of Spanish fluency than caregivers who were not interested in Spanish educational supports. Given the lack of relation between reported English fluency and interest in telepractice, these results suggest that telepractice was perhaps viewed primarily as a vehicle for increasing access to Spanish or bilingual speech and language services, rather than improving access to English services. The majority of the families reported that their children had never received any type of dual language support, despite being English learners, suggesting that the present participant sample generally had limited access to Spanish-speaking service providers.

Some caregivers reported that the limited access to bilingual services they experienced was difficult for them and their children, but others did not consider limited access to dual language services a problem. This finding is indicative of the broad spectrum of beliefs held by Spanish-speaking families. For some participants, maintaining Spanish proficiency was important; for others, achieving high levels of English proficiency was more important. The present research suggests that families’ values are critical in determining their preferred form of service delivery; there was a clear distinction between family language preference and the caregivers’ openness to telepractice service delivery.

### IMPLICATIONS FOR PRACTITIONERS

Important for clinicians who are considering using telepractice to facilitate increased access to service for families who speak minority languages, most of the survey respondents reported little-to-no prior knowledge of telepractice. This finding suggests that service providers may need to provide informational supports and resources regarding details of the service delivery model when presenting telepractice as an option to families. This point is highlighted by the families’ responses to the telepractice myths. Over 30% of survey respondents indicated that they believed that telepractice service delivery is not possible without owning a personal computer. Even more concerning, less than 20% of respondents were aware that telepractice is a legal form of service delivery. Given these findings, service providers may need to address these concerns when recommending telepractice to families. It may be beneficial to consider additional informational sharing of resources such as public service announcements or information about options to share at routine doctor’s visits or well-child checks.

### IMPLICATIONS FOR RESEARCH

Despite the small number of participants, the present sample was highly diverse. Families reported a substantial range of Spanish and English use in the home and varying levels of proficiency in each language. These findings lend support to calls for researchers to describe their samples of participants carefully, given the wide range of language environments in ELs’ homes. Considering that language exposure has been shown to be a key indicator of children’s academic performance (e.g., U.S. Dept. of Education, 2013), obtaining metrics of this exposure would appear to be critical to predicting outcomes accurately. Furthermore, metrics of both child and caregiver proficiency are important because the two are not consistently closely correlated.

## CONCLUSIONS

Results suggest that telepractice is a promising, but not yet widely accepted, service delivery model for young ELs. Families who have experienced barriers to needed services or who expressed interest in supporting their children’s Spanish language skills were more likely to be interested in telepractice, despite limited background knowledge about telepractice service delivery. It is recommended that practitioners provide thorough information about telepractice and its associated myths when considering telepractice as a service delivery option for families of ELs, and that practitioners work with families to identify priorities for their child’s care.

## Supplementary Material



## Figures and Tables

**Table 1:** Questionnaire Composite Indices

	Included Items			
**Caregiver Spanish Fluency**	Listening: Caregiver Spanish Fluency	Speaking: Caregiver Spanish Fluency	Reading: Caregiver Spanish Fluency	Writing: Caregiver Spanish Fluency
**Caregiver English Fluency**	Listening: Caregiver English Fluency	Speaking: Caregiver English Fluency	Reading: Caregiver English Fluency	Writing: Caregiver English Fluency
**Child Spanish Fluency**	Listening: Child Spanish Fluency	Speaking: Child Spanish Fluency		
**Child English Fluency**	Listening: Child English Fluency	Speaking: Child English Fluency		
**Value of Culture**	Importance of child being bilingual	Importance of child speaking Spanish	Importance of being bilingual in U.S.	
**Access to needed technology**	Access to a cordless phone	Access to a computer	Access to internet	Access to a web camera
**Competence with needed technology**	Competence with a cordless phone	Competence with a computer	Competence with internet	Competence with a web camera
**Belief in Telepractice Myths**	No computer-No telepractice	Child will not pay attention	Telepractice is lower quality	Telepractice is not legal

**Table 2:** Family Background Characteristics

Characteristic	%	*n*
**Country of Origin** (*n* = 78)		
Mexico	79.5	62
El Salvador	12.8	10
Other	7.7	6
**Geographic Location** (*n* = 61)		
Florida	67.2	41
Illinois	21.3	13
Michigan	9.8	6
Other	1.6	1
**Experience with Bilingual Services** (*n* = 69)		
None	75.4	52
Some bilingual services	24.6	17
**Interest Spanish support for Child?** (*n* = 68)		
No	44.1	30
Yes	55.9	38
**Child Birthplace** (*n* = 77)		
United States	90.9	70
Non-United States	9.1	7
**Language Child Speaks at Home** (*n* = 75)		
Spanish	54.7	41
More Spanish than English	16.0	12
Balanced Spanish/English	26.7	20
More English than Spanish	1.3	1
English	1.3	1
**Speech/Language Disorder Severity** (*n* = 23)		
Mild	30.4	7
Moderate	60.9	14
Severe/Profound	8.7	2

**Table 3:** Correlation Coefficients

	1	2	3	4	5	6	7	8	9
1. Interest in receiving telepractice	1								
2. Caregiver Spanish fluency	.31**	1							
3. Child diagnosis of speech/language disorder	.47**	.06	1						
4. Caregiver interest in receiving Spanish support	.62**	.31**	.36**	1					
5. Caregiver English fluency	−.04	.04	.19	.26*	1				
6. Child Spanish fluency	−.01	.42**	−.18	.04	.01	1			
7. Child English fluency	−.22	−.03	−.23*	−.27*	−.03	.11	1		
8. Caregiver value of culture	.03	.21*	−.09	−.09	−.06	.24*	.15	1	
9. Access to Technology	.10	.10	.20	.18	.32**	.08	.05	.07	1
10. Competence with Technology	.12	.03	.25*	.22	.37**	−.11	−.06	.04	.67**

* Significant at *p* < .05

** Significant at *p* < .01

**Table 4:** Logistic Regression Predicting Interest in Telepractice

Model including three predictors					

	B	SE	Wald	Sig.	Exp (B)
Diagnosed disorder	2.40	.87	7.65	.006	11.03
Caregiver fluency in Spanish	.60	.55	1.17	.278	1.82
Interest in Spanish support	−2.33	.83	7.78	.005	.10
Constant	−.24	.57	.18	.671	.78
Model χ^2^ =	33.47 (*p* <.001)				
Pseudo R^2^ =	.604				
*n* =	55				

Model including two predictors					

	B	SE	Wald	Sig.	Exp (B)

Diagnosed disorder	2.35	.84	7.86	.005	10.49
Interest in Spanish support	−2.70	.79	11.53	.001	.07
Constant	.07	.48	.02	.876	1.08
Model χ^2^ =	34.40 (*p* <.001)				
Pseudo R^2^ =	.585				
*n* =	60				

*Note*. Categorical variables were coded as follows: not interested in telepractice = 0; interested in telepractice = 1; no diagnosis or unsure = 0; diagnosed disorder = 1; not interested in Spanish support = 0; interested in Spanish support = 1.
