# Supplementary material for: The Social Validity of Telepractice among Spanish-Speaking Caregivers of English Learners: An Examination of Moderators
Source: Int J Telerehabil. 2017 Nov 20;9(2):13–24. doi: 10.5195/ijt.2017.6227 (PMC5716612; doi:10.5195/ijt.2017.6227)
Supplement: Supplementary file 1 [file ijt-09-13-s01.pdf]

International Journal of Telerehabilitation 10.5195/ijt.2017.6227 –Supplemental File

Child age: ________________________________

Relation to child: ________________________________

Current city and state: ________________________________

What country (or state) is your family from? ________________________________

Where was your child born? ________________________________

1. Please indicate: How many years of school have you had?

- ☐ Elementary school
- ☐ Some high school
- ☐ Graduated high school
- ☐ Some college
- ☐ Graduated college
- ☐ Finished graduate school
- ☐ Other - please specify: _______________

2. Please mark the box that indicates: What is **your** fluency level **in**…

…Spanish?

	None	Minimal	Passable	Adequate	Strong	Fully fluent
**Listening**						
**Speaking**						
**Reading**						
**Writing**						

…English?

	None	Minimal	Passable	Adequate	Strong	Fully fluent
**Listening**						
**Speaking**						

**Reading**						
**Writing**						

3. What is your **child's** fluency level **in…**

**…Spanish?**						
	None	Minimal	Passable	Adequate	Strong	Fully fluent
**Listening**						
**Speaking**						

**…English?**						
	None	Minimal	Passable	Adequate	Strong	Fully fluent
**Listening**						
**Speaking**						

4. How important is it to you...

	**Not important**	**A Little important**	**Moderately Important**	**Considerably Important**	**Very important**
**...for your child to be bilingual?**					
**...for your child to speak Spanish?**					
**...for your child to speak English?**					
**...to participate in your child's schooling?**					
**... to be bilingual in the United States?**					
**... to speak English in the United States?**					

5. Have you heard of telepractice (distance therapy)?

Yes / No

If yes, please explain:____

6. "Telepractice" is the **use of technology to deliver services**, whether medical, educational, etc., **remotely**. One example of "telepractice" is a psychologist having a counseling session with a client over the phone. Another example is a speech therapist providing therapy over Skype. Telepractice can involve a variety of technologies, such as phone, Facetime, and videoconferencing.

Telepractice can also be used to provide intervention to children. Intervention can be provided directly to the child or to a caregiver to support the child's learning. For example, a therapist could give directions and coach a caregiver to provide therapy to a child. This form of intervention is a very effective strategy according to much research. If your child were to need therapy services, **would you be interested in him/her receiving services via telepractice**? Why or why not?

Yes / No

Why or why not?:____

7. Imagine this situation: you live an hour away from a big city. You recently discovered that your child has a disorder and needs help from a tutor or speech therapist. In the city, there is a bilingual therapist that offers telepractice services over videoconferencing that your child could receive in your home. The bilingual therapist also offers in-person services in the city. In your town, there is a therapist that only speaks English. **Which option will you choose**? All the options cost the same.

- ☐ The bilingual therapist's services in person in the city
- ☐ The bilingual therapist's services over telepractice
- ☐ The bilingual therapist's services half in person and half over telepractice
- ☐ The services of the therapist who speaks English

8. In your opinion, what are the benefits of receiving services via telepractice?

9. Please indicate the level of access you have to the following technologies:

	No access	Limited access	Reliable access	Unlimited access
**Cordless Phone**				
**Computer**				
**Internet**				
**Web Camera**				

10. Please indicate the level of ability/comfort you have with the following technologies:

	No ability	Limited ability	Moderate	Much ability
**Cordless Phone**				
**Computer**				
**Internet**				
**Web Camera**				

11. Please indicate your agreement with the following phrases:

a) I don't have a computer. I can't receive services by telepractice.

- ☐ I completely agree
- ☐ I more or less disagree
- ☐ I more or less agree
- ☐ I disagree
- ☐ I am unsure

b) My child has no interest in technology and won't pay attention to telepractice services.

- ☐ I completely agree
- ☐ I more or less disagree
- ☐ I more or less agree
- ☐ I disagree
- ☐ I am unsure

c) Telepractice services are not as good as services in person.

- ☐ I completely agree
- ☐ I more or less disagree
- ☐ I more or less agree
- ☐ I disagree
- ☐ I am unsure

d) Telepractice isn’t legal.

- ☐ I completely agree
- ☐ I more or less disagree

☐ I more or less agree

☐ I disagree

☐ I am unsure

12. What goals do you have for your child in school?

____

____

____

13. Have you received any information from anyone regarding intervention in Spanish versus English? If yes, what information have you received? Who gave you the information?

Yes / No

____

____

14. Ideally, who would deliver services to your child? In what language would he/she speak?

Preferred language: ________________________________

Explanation:____

____

15. Please mark the boxes to indicate in what language...

	**Spanish**	**More Spanish and less English**	**Spanish and English**	**More English and less Spanish**	**English**
**...do you speak at home?**					
**...does your child speak at home?**					
**...does your child speak with others?**					
**... do you speak with your child at school?**					
**... do you speak with your child at the supermarket?**					

16. Please indicate by circling: Does your child have a diagnosed speech or language disorder or receive special services at school?

Yes (**continue to the next page)**

No (**end questionnaire)**

If unsure – **end questionnaire**

<u>Questions for families of children with diagnosed speech/language impairment:</u>

17. Has your child received services of a speech-language pathologist (or speech therapist) before today? If your child has received services, what language were the services delivered in?

Yes / No Language: ____________________

18. Have you had difficulty obtaining services of a bilingual therapist? If yes, what difficulties have you experienced?

Yes / No

____

____

19. According to you, what is your child's principal speech and language problem?

____

____

20. Please mark the box to indicate the severity of your child's speech/language disorder.

Very mild	Mild	Mild-moderate	Moderately-severe	Severe	Very severe/profound

21. Would you be interested in your child receiving speech and language intervention in Spanish?

Yes / No

Thank you for participating!
